# Systematic *in vivo* evaluation of the time-dependent inflammatory response to steel and Teflon insulin infusion catheters

**DOI:** 10.1038/s41598-017-18790-0

**Published:** 2018-01-18

**Authors:** Jasmin R. Hauzenberger, Julia Münzker, Petra Kotzbeck, Martin Asslaber, Vladimir Bubalo, Jeffrey I Joseph, Thomas R. Pieber

**Affiliations:** 1 Department of Internal Medicine, Division of Endocrinology and Diabetology, Medical University of Graz, Graz, Austria; 2 Institute of Pathology, Medical University of Graz, Graz, Austria; 3 Division of Biomedical Research, Medical University of Graz, Graz, Austria; 4 Department of Anesthesiology, Jefferson Artificial Pancreas Center, Thomas Jefferson University, Philadelphia, PA USA

## Abstract

Continuous subcutaneous insulin infusion (CSII) catheters are considered the weak link of insulin pump therapy. Wear-time considerably varies between patients and the choice of catheter material is based on personal preferences rather than scientific facts. Therefore, we systematically assessed and quantified the inflammatory tissue response to steel versus Teflon CSII catheters over a maximum wear-time of 7 days in swine. Tissue surrounding catheters was analysed using histopathology and quantitative real-time PCR. The area of inflammation increased significantly over time independent of material which was confirmed by an increase in CD68 expression and an increase in mononuclear and neutrophil cell infiltrate around the catheters. We observed substantially higher fibrin deposition (p < 0.05) around steel on day 4 of wear-time. IL-6 gene expression increased within 24 hours after insertion, returned to normal levels around Teflon (p < 0.05) but remained high around steel (p < 0.05). IL-10 and TGF-β levels did not resolve over time, indicating impaired wound healing. In conclusion, there was a major temporal effect in the acute inflammatory response to CSII catheters but we found little difference between materials. This study setup presents a robust tool for the systematic analysis of the tissue response to CSII catheters.

## Introduction

Continuous subcutaneous insulin infusion (CSII) catheters are the most crucial part of insulin pump therapy for insulin dependent patients^1–5^. They have been on the market since the late 1980s and are manufactured with both Teflon (polytetrafluoroethylene) and steel cannulas^6,7^. The choice of one material over the other is largely based on the patient’s personal preference, his or her endocrinologist’s or diabetes educator’s opinion and therapy costs^8–10^. There is a trend in both the United States and Europe towards using Teflon sets (90% and 75%, respectively) but approximately 40 to 45% of pump users in Germany use steel catheters^4,8,11^. Compared to Teflon, steel catheters are easier to insert and are less prone to kinking, and can be worn by patients allergic to Teflon. Patients using steel catheters report better metabolic control, less variable insulin absorption and less unexplained hyperglycemia^8,12^. However, especially during exercise, steel may cause discomfort and the softer and more flexible Teflon catheter is assumed to be more comfortable to wear^8,13,14^. The wear-time of the CSII catheter considerably varies between patients (from 2 to 10 days), although recommendations for the optimal frequency of changing an insulin infusion set (2 days for steel and 3 days for Teflon) exist^3,9,15^. Independent of material, the introduction of a cannula into the subcutaneous adipose tissue elicits an inflammatory response. The degree of inflammation and tissue response, however, depends on the cannula material properties, including stiffness or rigidity, surface nanostructure and the cannula shape, e.g. presence of a sharp tip^16–18^. Interestingly, most studies on CSII catheter tolerability, complications and wear-time are based on patient questionnaires and are thus assessing mainly subjective data^2,3,15,19,20^. Studies are lacking that assess the inflammatory tissue response as determinant on material-tolerability and optimal wear-time of CSII catheters in order to scientifically underline the choice of one material over the other. To close this gap, we systematically evaluated the inflammatory subcutaneous adipose tissue response to the steel and Teflon cannulas of commercially available CSII catheters over 7 days of wear-time.

## Research Design and Methods

All animal experiments were performed according to Austrian law and ethical regulations. The animal study including 10 female farm swine (*sus scrofa domesticus*) was approved by the Austrian Federal Ministry of Science, Research and Economy and performed in consent with Directive 2010/63/EU on the protection of animals used for scientific purposes (BMWFW Gz).

### Animal Care

The animals arrived at the animal facility (Institute for Biomedical Research, Medical University of Graz, Austria) 7–10 days prior to the first study day for acclimatisation. Animals were housed in groups of 2 per corral before study start and separately after insertion of the first catheters. The corral size provided at least 2.5 m² per animal and was equipped with enrichment devices (bale of straw, balls and rubber rings). The animals’ well-being, health condition and the catheter sites were checked regularly.

### CSII Catheters

We used 2 different commercially available CSII catheters (Medtronic MiniMed, Northridge, CA). A CSII catheter consists of a plastic hub or connector which is attached to the skin via an adhesive, and a cannula that is inserted into the subcutaneous adipose tissue. For insulin infusion an insulin pump can be connected to the hub via plastic tubing and insulin will be delivered into the subcutaneous tissue through the cannula. The Medtronic MiniMed™ Sure-T™ insulin infusion set has a 6 mm 29 gauge steel cannula with a sharp, slanted bevel tip to allow manual insertion. The Medtronic MiniMed™ Quick-set™ has a 6 mm 25 gauge Teflon cannula with a blunt end and is introduced into the subcutaneous tissue via a 27 gauge introducer steel needle with sharp tip. After insertion, the introducer needle is removed and discarded.

### Insertion of Catheters

The study duration was 8 days. On days 1, 4 and 7, catheters were inserted to assess wear-time of 7 days (insertion on day 1), 4 days (insertion on day 4) and 1 day (insertion on day 7) in the same animal (Table 1). On day 1 of the study (wear-time = 7 days), swine were anesthetised and shaved on the back and the flanks. Skin was washed with soap and water, disinfected and covered with a layer of clear medical adhesive, to ensure better adhesion of the catheters and avoid loss of catheters due to movement or friction. Four catheters were implanted (2 Sure T™, 6 mm steel and 2 Quick-set™, 6 mm Teflon). An insulin pump (Medtronic MiniMed Paradigm™) containing a saline filled reservoir was connected to each hub of the Quick-sets directly after insertion. A bolus of 3–4 units (30–40 µl) of saline was infused to ensure that none of the catheters had kinked during insertion. In case of kinking, pressure in the cannula increases and the insulin pump produces an occlusion alarm. After ensuring that the cannulas had been properly inserted into the tissue, the tubing was disconnected, the hub closed with a cap and covered with a small piece of medical gauze. Another layer of medical adhesive was applied, followed by Kinesiology tape to firmly secure the catheters and hold them in place over the duration of the study. A stockinet was pulled over the animals and 4 holes cut out for the legs. Sharp edges were padded with cotton. On days 4 (wear-time = 4 days) and 7 (wear-time = 1 day), animals were sedated, the stockinet removed and additional CSII catheters (4 each day) inserted. Catheters were covered with clear medical adhesive and Kinesiology tape, and the stockinet put back on. On days 2, 3, 5, and 6, animals were able to move freely. Animals were euthanised before tissue excision and after methylene blue dye infusion on day 8. In total, 120 catheters were inserted (60 steel and 60 Teflon). Half of the samples were dedicated for histopathological analyses and the other half for quantitative real-time PCR. A piece of non-traumatised reference tissue was excised for messenger RNA analysis and for calculation of relative expression fold change.

**Table 1:** Study design; n = 10 animals.

*Study day no*.	1 (Fri)	2 (Sat)	3 (Sun)	4 (Mon)	5 (Tue)	6 (Wed)	7 (Thu)	8 (Fri)
*Catheters*	4			4			4	Tissue explantation
*Wear time*	7 days			4 days			1 day	

### Excision and Histology

On study day 8 the stockinet and the medical adhesive were carefully removed to avoid pulling out catheters. Teflon catheters were infused with 4 units (40 µl) of methylene blue surgical dye, using an insulin pump to check for occlusion alarms caused by kinked catheters, catheter obstruction or morphological changes in the subcutaneous tissue. A fatty tissue specimen (approximately 5 × 5 × 2 cm) was removed from around the catheters and placed in 4% PBS-buffered formaldehyde for 72 hours with the CSII catheter still remaining in the tissue. The catheter was removed right before grossing the tissue specimen to locate the insertion channel and the surrounding region of interest. The specimen was dehydrated in Formalin for 2 hours, followed by a rising ethanol series over 6 hours at 40 °C and embedded in paraffin wax. Four micrometer thick paraffin-tissue sections were stained with Hematoxylin and Eosin (H&E) and Masson’s Trichrome following standard protocols. Sectioning was repeated 3 times for each sample with approximately 9 µm spacing. The first section confirmed the location of the insertion channel. The consecutive sections represented deeper layers of the tissue (i.e. areas more distant from the center of the insertion channel). All slides were scanned with Aperio ScanScope AT and analysed using the ImageScope Version 12 software (both: Aperio Technologies, Inc., Leica Biosystems). On whole slide images, the area of inflammation (mm^2^), fibrin deposition (mm^2^) and fat necrosis (mm^2^) around the insertion channel was measured by the pathologist. The distance from epidermis to lowest point of observed inflammation was measured (mm). Furthermore, the area of chronic inflammatory reaction (lymphocytes and monocytes) and the density of diffuse infiltrates of neutrophil granulocytes was evaluated and categorised as *none*, *some*, *mild*, *moderate* or *severe* (0, 0.5, 1, 2, 3). Slides were analyzed twice to exclude an intra-observer bias. After all sections of each sample had been analysed, we considered only the largest value of the 3 sections per sample (=maximum trauma observed in all tissue sections) for further calculation. Unblinding and statistical analyses were performed after the histopathological evaluation was completed.

### Quantitative Real-time PCR

For RNA isolation, the tissue plug was grossed down to about 5 mm distance from the cannula. The cylinder-shaped specimen was separated into 2 different sections along the cannula (subcutaneous region). Tissue was placed in an RNA stabilising solution (RNA*later*, QIAGEN, Germany) at 4 °C for at least 24 hours before isolating the RNA (RNeasy Mini Kit, QIAGEN, Germany). RNA was transcribed to cDNA (Applied Biosystems High Capacity cDNA Reverse Transcriptase kit, Thermo Fisher Scientific, Austria) and quantitative real-time PCR was performed using the SYBR Green method (LightCycler® 480 SYBR Green I Master, Roche, Austria) for animal samples. We analysed expression of macrophage marker CD68 and the cytokines interleukin-6 (IL-6), interleukin-8 (IL-8/CXCL8), tumor necrosis factor alpha (TNF-α), transforming growth factor beta (TGF-β) and interleukin-10 (IL-10). All qPCRs were run on a Roche 480 Light Cycler® system. Relative expression fold change was calculated using the ddCt method with 2 reference genes, namely beta-Actin (ACTB) and tyrosine 3-monooxygenase/tryptophan 5-monooxygenase activation protein zeta (YWHAZ) according to the MIQE guidelines^21^. Expression was corrected for primer efficiency and is shown relative to gene expression in non-traumatised tissue (no catheter).

### Statistical Analyses

Continuous data (e.g. cytokine gene expression) were examined for normality using Shapiro Wilks tests with Lilliefors significance correction as well as by visual data inspection using Q-Q plots. Catheter material (steel versus Teflon) as well as catheter wear time (1, 4, and 7 days) and the interaction term were included as within subject factors in generalised linear mixed models (GLMM) with a log normal distribution for longitudinal non-Gaussian data (e.g. IL6, IL8, IL10 or TNF-alpha) and identity link function for normally distributed data (distance to lowest point of inflammation). The model selection process to define the appropriate covariance structure (first order autoregressive, compound symmetry, diagonal or unstructured) was based on Akaike’s Information Criterion (AIC), an index of relative goodness-of-fit to model the subject variation. The smaller the information criteria value, the better the model fits, i.e. the model that minimized AIC was preferable. To compare different covariance structures for the same fixed effects model, we used the change in 2 REML logL for an approximate Likelihood Ratio Chi-Square test additionally.

Generalised estimation equations (GEE) models with an exchangeable (compound symmetry) correlation structure and a cumulative logit link-function were used for ordinal data (e.g. grade of cellular infiltration) to specify differences between catheter materials over wear time.

All analyses were based only on available data without imputation. P-values were adjusted for multiple testing with Bonferroni correction. Resulting adjusted p values less than 0.05 were considered significant. All statistical tests were performed using SPSS version 23.0 (SPSS Inc., Chicago, IL) and GraphPad Prism version 5.0 (GraphPad Software, San Diego, USA) for visualisations.

## Results

**Figure 1:**
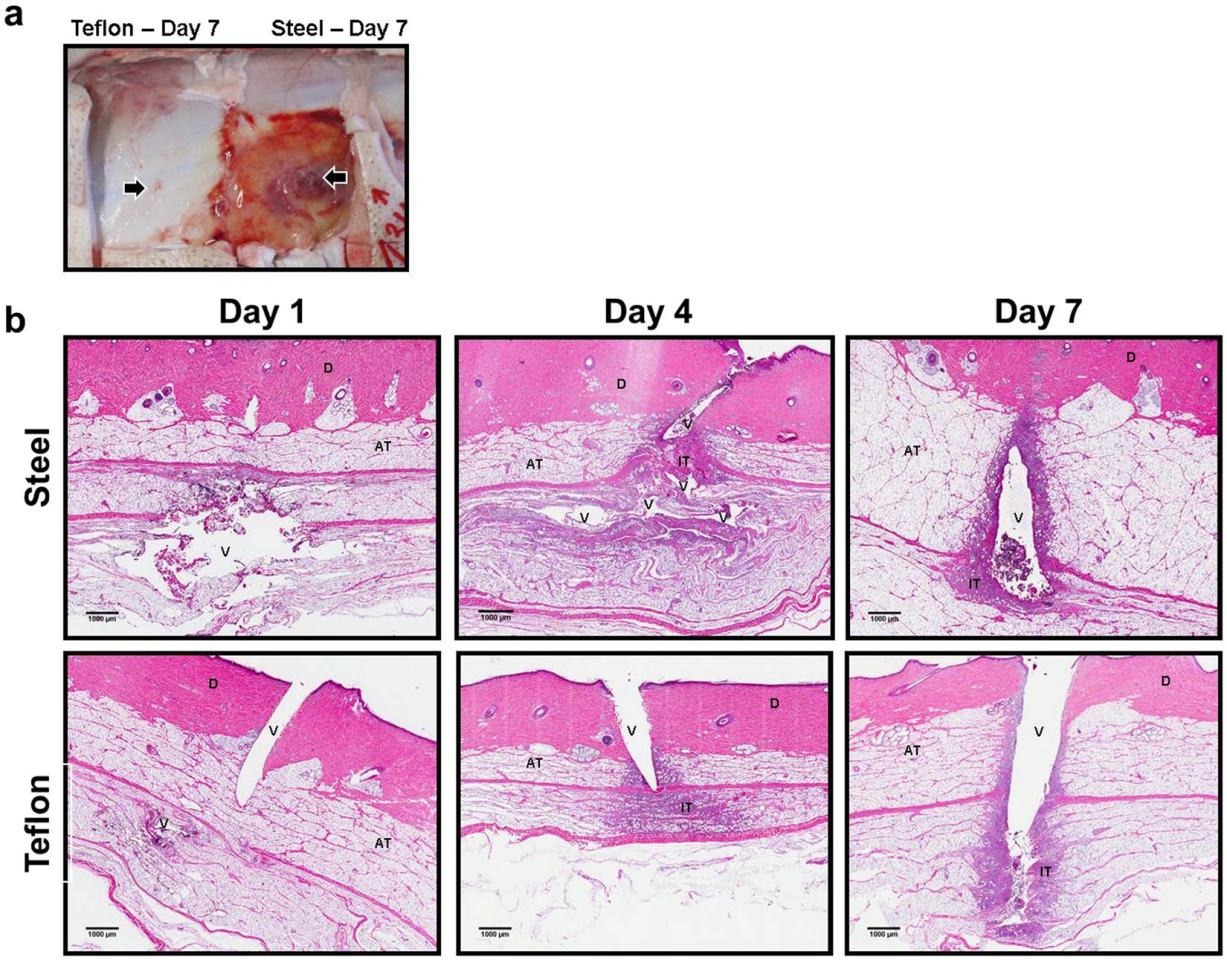
Tissue after excision and representative histology slides. (**a**) Fascia layer photographed after removal of square specimens of skin and adipose tissue around CSII catheters after 7 days of wear time (left: Teflon, right: steel). Arrows are pointing at locations where tip hit the muscle. Tissue is severely inflamed below the sharp tip of the steel cannula. (**b**) Examples of tissue sections stained with hematoxylin & eosin (20 × magnification) revealing the void (V) left by steel (top row) and Teflon (bottom row) cannulas in the dermis (D) and subcutaneous adipose tissue (AT). The inflammatory tissue (IT) is mainly characterised by mononuclear inflammatory cells migrating towards the site of trauma and central fibrin deposition that form a layer around the insertion channel.

Throughout the study, 120 CSII catheters were inserted. 4/120 cannulas (2 steel and 2 Teflon) kinked above the medical adhesive covering the animal skin. Insertion channels after excision and grossing of specimens were located in 115 specimens. In total, 9 samples were rejected (failed insertion or no insertion channel located), leading to a dropout rate of 7.5%. The infusion of saline into the Teflon catheters after insertion did not result in a single occlusion alarm or leakage. Infusion of methylene blue dye into the 30 Teflon catheters used for histology prior to excision resulted in 3 “no delivery” occlusion alarms by the Paradigm™ pump (10%). Tissue specimens were excised down to the fascia planes that exhibited nearly no inflammation below Teflon cannulas but revealed substantial fibrosis and inflammation below steel cannulas inserted for 4 days or 7 days (Fig. 1a). Figure 1b shows representative examples of subcutaneous adipose tissue stained with H&E after the removal of the CSII catheters. We could observe substantial tissue disruption by the movement of a stiff steel cannula (Fig. 1b, top row) while the void left by Teflon was consistent with the cylindrical shape of the cannula (Fig. 1b, bottom row). Inflammatory cells (stained dark purple) migrated towards the site of trauma and, by day 7, formed a layer around the foreign body.

**Figure 2:**
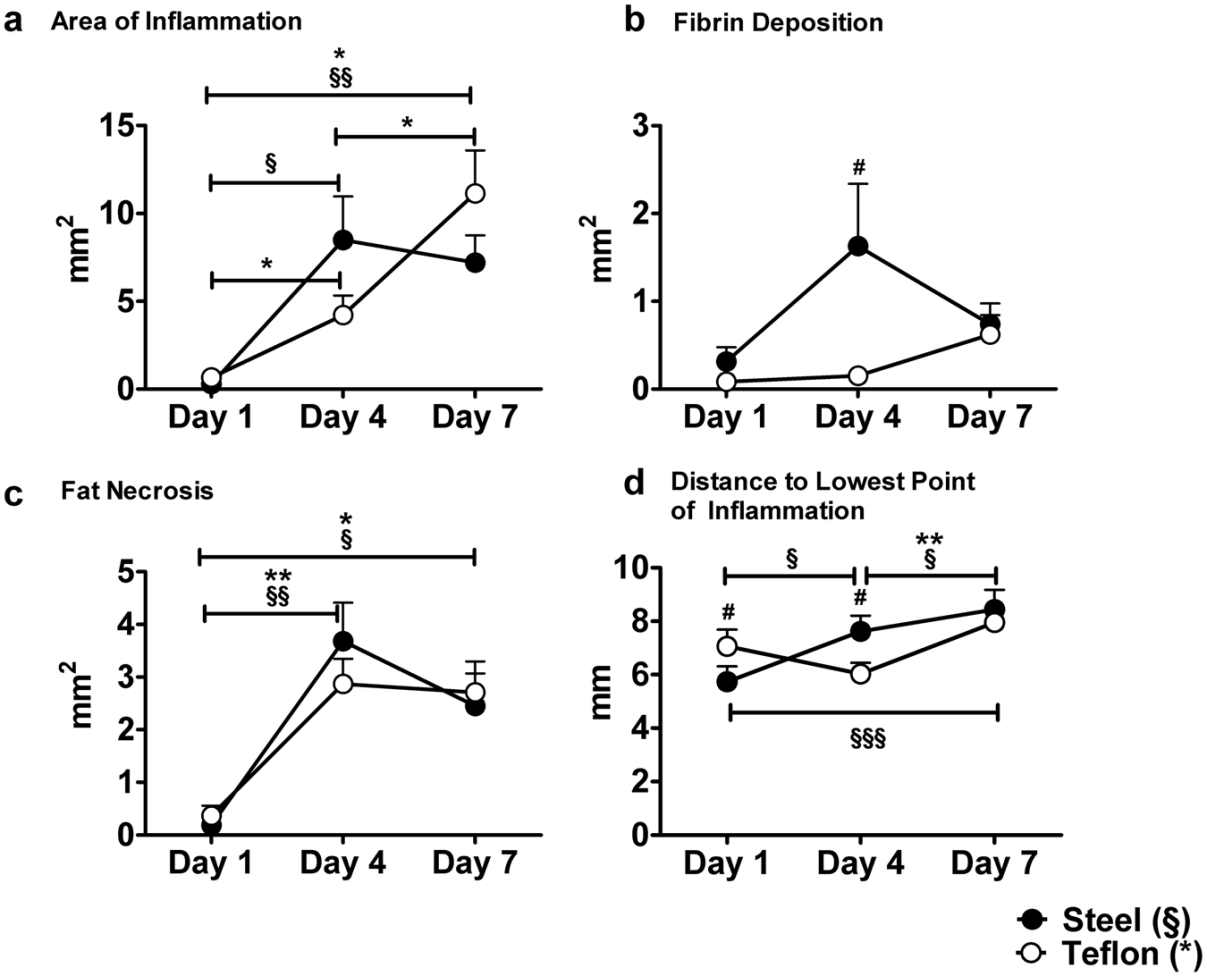
Trend curves for histopathological characteristics over 7 days of wear time. (**a**) Area of inflammation = total area of disrupted and inflamed tissue, including inflammatory cells, fat necrosis and fibrin deposition; (**b**) Fibrin deposition = area of fibrin in the immediate vicinity of the cannula; (**c**) Fat necrosis = area of destroyed fat cells in the immediate vicinity of the cannula; (**d**) Distance to lowest point of inflammation = distance from skin surface to the last point of observed inflammatory tissue in an approx. 90° angle. The cannula length was 6 mm. (***^/§§§^p < 0.0001; **^/§§^p < 0.005; *^/§^p < 0.05; ^#^p < 0.05 difference between materials; note the different scales on y-axis. Data are presented as mean ± SEM).

The area of inflammation, measured by the pathologist, increased significantly around both materials between day 1 and day 4 as well as day 1 and day 7. The area of inflammation around the steel catheter plateaued after 4 days but further increased around Teflon (p < 0.05; Fig. 2a). We could detect no temporal effect for fibrin deposition around steel catheters and only a trend around Teflon catheters (p = 0.097 between day 1 and day 4, p = 0.059 between day 1 and day 7). We observed a significantly larger area of fibrin around steel catheters on day 4 (p = 0.029, Fig. 2b). The area of necrosed fat cells was significantly increased on days 4 (p = 0.001 for Teflon, p = 0.002 for steel) and 7 (p = 0.005 for Teflon, p = 0.009 for steel) compared to day 1 with no difference between day 4 and day 7 (Fig. 2c).

Both cannulas had a length of 6 mm but Teflon cannulas were inserted into the skin using a slightly longer introducer steel needle with a sharp tip. Therefore, the distance to the lowest point of observed inflammation was longer for Teflon catheters on day 1 (7.1 mm versus 5.7 mm; p = 0.033; Fig. 2d). This initial trauma caused by the introducer needle decreased until day 4 (6.0 mm; n.s.) while the inflammation distance from the epidermis increased for steel catheters in that time (p = 0.007). The distance further increased between day 4 and day 7 to 8.4 mm along steel and 8.0 mm along Teflon catheters (p = 0.048 and p = 0.001, respectively). The distance to lowest point of observed inflammation was significantly higher for steel than for Teflon on day 4 of wear time (p = 0.019) but similar on day 7.

**Figure 3:**
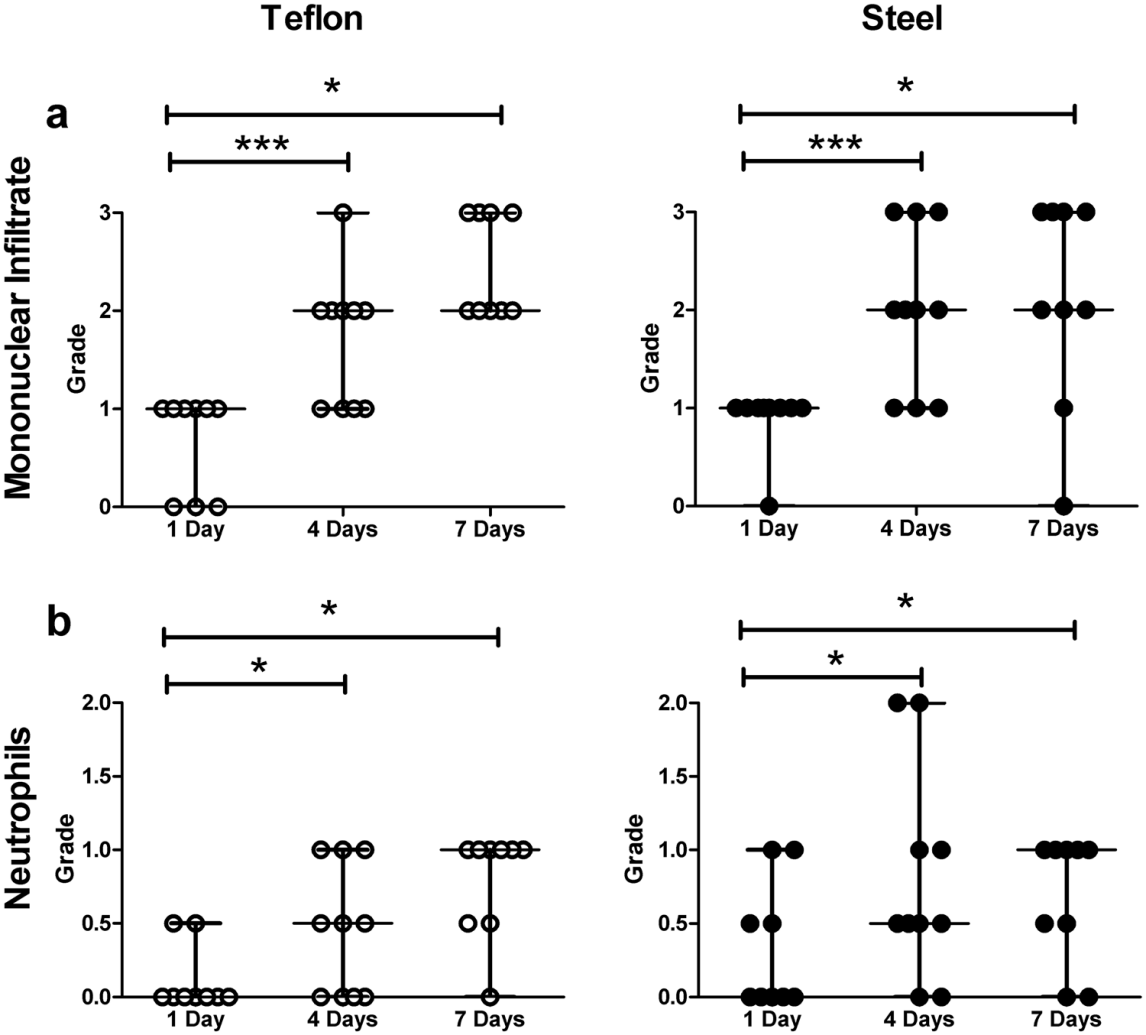
Results of ordinal grading of the density of (**a**) mononuclear cells ( = lymphocytes, monocytes, neutrophils) and (**b**) neutrophils around the insertion channel. The grades on the y-axis are as follows: 0 = none; 0.5 = some; 1 = mild; 2 = moderate; 3 = severe. (*p < 0.05; **p < 0.005; ***p < 0.0001; data are presented as median and range).

Qualitative grading of density of inflammatory cells (*none*, *some*, *mild*, *moderate*, *severe*) around the insertion channel was not significantly different between materials. However, density of mononuclear infiltrate and neutrophils was significantly elevated on day 4 (p < 0.0001 and p = 0.02, respectively) and day 7 (p = 0.01 and p = 0.03, respectively) compared to 24 hours of wear-time (Fig. 3a and b).

**Figure 4:**
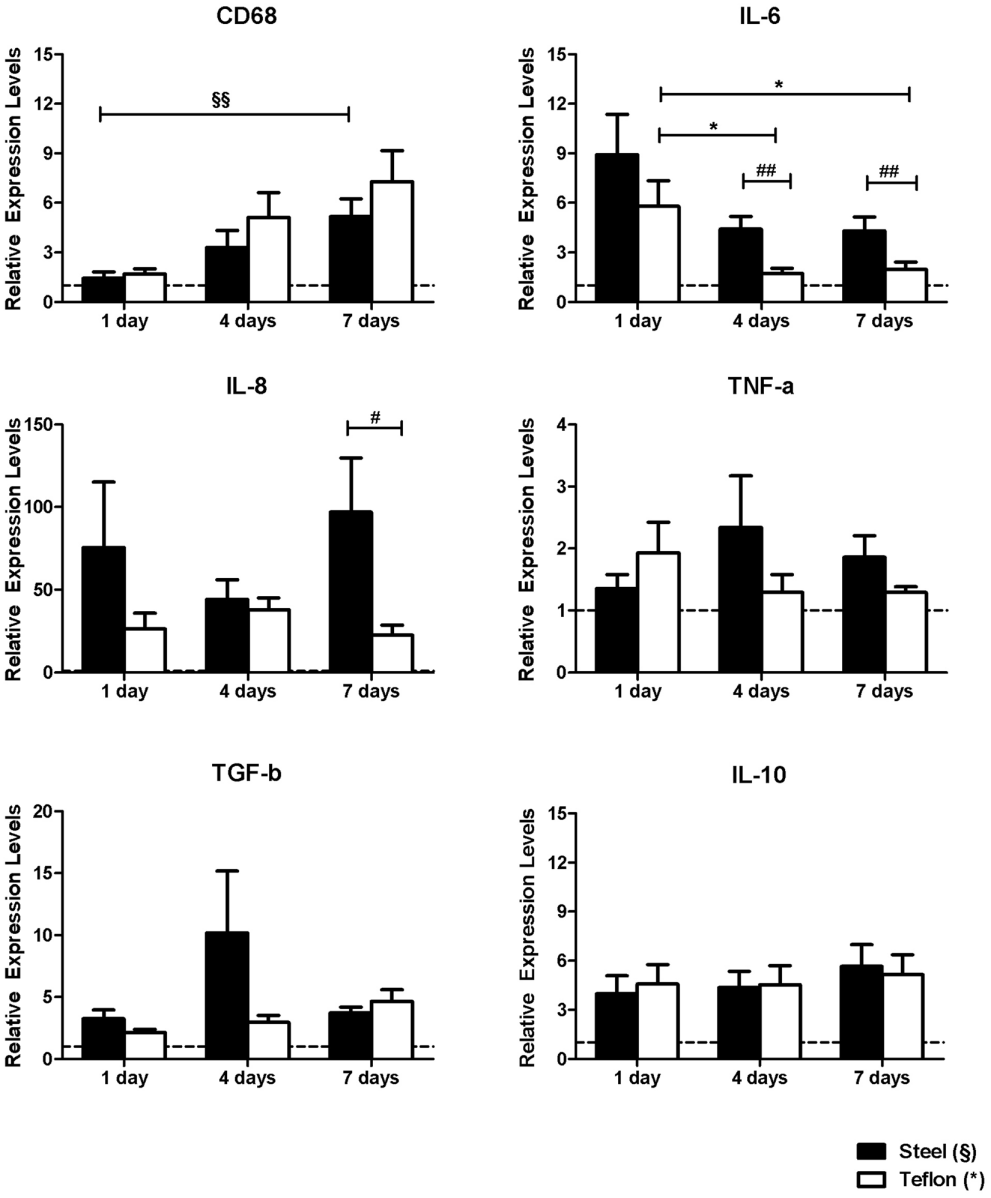
Relative expression fold change of CD68 macrophage marker and pro- and anti- inflammatory cytokines in the vicinity of the catheter over 7 days of wear time. The intermitted line represents 100% expression under non-traumatic conditions (**^/§§^p < 0.005, *^/§^p < 0.05; ^##^p < 0.005 and ^#^p < 0.05 difference between materials; note the different scales on y-axis. Data are presented as mean ± SEM).

Quantitative real-time PCR are shown in Fig. 4. We detected an increase of macrophage marker CD68 gene expression between 1 day and 7 days of wear-time for both steel ((1.4 ± 1.2-fold vs. 5.2 ± 3.4-fold; p = 0.006) and Teflon (1.7 ± 0.9-fold vs. 7.2 ± 5.6-fold; p = 0.065). We could not identify a significant difference between materials on either day. After 24 hours, there was almost no change in CD68 expression independent of material. IL-6 expression was highest on day 1 and decreased significantly around Teflon over 4 and 7 days of wear-time (both p = 0.034). IL-6 levels were significantly higher around steel than Teflon after 4 days (p = 0.005) and after 7 days (p = 0.047) of wear-time. Mean IL-8 levels were higher for steel on day 7 of wear time (p = 0.029).

TNF-α gene expression increased to a maximum of 2-fold expression around both materials but there was no statistical effect detected over time or between materials. TNF-α expression was slightly higher around steel compared with Teflon on day 7 (p = 0.083). TGF-β levels were not significantly different over time and between materials. TGF-β gene expression levels around steel showed a rather high variability on day 4 with values ranging from 0.9-fold to 49.7-fold among samples (mean 10.2 ± 5.0-fold). There was a trend towards an increase in expression of TGF-β around Teflon catheters between day 7 and day 1 of wear-time (p = 0.058).

Anti-inflammatory IL-10 gene expression increased approximately 4-fold within the first 24 hours of wear time and remained stable and at similar levels around both materials throughout the week of wear-time.

## Discussion

We performed a systematic *in vivo* evaluation of the inflammatory response to commercially available CSII catheters to identify determining factors for tolerability of steel versus Teflon.

Independent of material, the insertion of a CSII catheter causes the disruption of capillaries leading to fibrin and platelet deposition^22^. Proteins non-specifically adsorb to the material surface and neutrophils, platelets and monocytes form a layer around the foreign body, releasing pro-inflammatory cytokines to recruit more white blood cells and to initiate the differentiation of monocytes into macrophages^22–26^.

Our results suggest that small movement of the sharp tip of the steel cannula continuously damaged microvasculature in swine adipose tissue which results in significant higher fibrin deposition after 4 days of wear-time and a significant difference in trauma depth in the subcutaneous tissue. Studies show that the shape of the implanted material has a major effect on tissue reaction and macrophage attachment, suggesting that round shapes elicit a less severe response than shapes with sharp edges^17,27,28^. However, the overall area of inflammation and fat necrosis did not differ between materials and the only effect observed was attributed to wear-time rather than material. The increase in area of inflammation over wear-time could be confirmed by grading the density of inflammatory cells which was also not different between steel and Teflon. Additionally, these results were confirmed by an increase in macrophage gene expression over time.

Several published *in vivo* and *in vitro* studies exist, regarding the recruitment and attachment of inflammatory cells to steel or Teflon but are sometimes contradicting and difficult to interpret^29,30^. While a study by Bussutil *et al*. showed significantly less macrophage and neutrophil adhesion to intraperitoneally implanted steel disks^29^, Hallab *et al*. claim a five-fold increase in adhesion strength of macrophages to steel surfaces compared to Teflon^30^. Furthermore, it has been shown that a Even though the Teflon cannula was thicker, had a larger inner diameter and thicker walls than the steel cannula (29 versus 25 gauge), it did not elicit a less severe tissue reaction. The area of inflammation even increases significantly between day 4 and day 7, while it remains unchanged around steel after 4 days.

The stiffness of the material can also determine the extent of trauma, with sheer forces continuously damaging the adipose and connective tissue in the cannula’s vicinity and is a major influential factor in inflammatory cell activation^18,31^. Softer materials elicit less scaring by decreasing the strain on the tissue^18^.

This cell activation further depends on the release of pro-inflammatory cytokines, such as IL-6, IL-8 and TNF-α by macrophages and other cells adherent to the cannula^25,32^. Macrophages are said to respond to biomaterials immediately via IL-6 secretion^33^. Our results show that the secretion of IL-6, which is associated with reepithelisation and scarring^34^, is increased approximately 9-fold (steel) and 6-fold (Teflon) within 24 hours after initial trauma and decreases significantly with wear-time. Interestingly, IL-6 gene expression levels are significantly higher around steel cannulas than around Teflon cannulas on days 4 and 7. Levels in tissue surrounding Teflon return to values comparable with non-traumatised adipose tissue by day 4. A possible explanation for this could be that the sharp tip of the steel cannula continuously injures the tissue throughout wear time. Concordant with literature^35^, we showed that the major chemotactic factor IL-8/CXCL8 is highly expressed within 24 hours after cannula insertion. High levels of IL-8 are known to be associated with impaired wound healing, which is the case when a foreign body is present^36^. We showed that after 7 days, steel cannulas elicit an approximately 100-fold increased IL-8 expression and a 23-fold increase caused by Teflon, indicating that independent of material, the presence of a foreign body itself leads to unresolved inflammation. Immediately after trauma, TNF-α production is upregulated to enhance phagocytosis^22^. In our results, this is the case for both materials although in average the induction was minor compared with results from other studies. Pachler *et al*., for instance, showed a 25-fold increase in TNF-α protein levels within 8 hours after inserting a Teflon open-flow microperfusion probe subcutaneously^37^. However, the group perfused the probe continuously which may alter the resulting inflammatory response.

During wound healing, macrophages undergo a phenotype switch and release anti-inflammatory IL-10 and TGF-β^32,38^. TGF-β release activates fibroblasts which start to synthesize collagen to lay down new extracellular matrix^32^. In our study, TGF-β gene expression levels are immediately upregulated within the first 24 hours of wear time and while they steadily increase around Teflon cannulas, their levels around steel peak on day 4 of wear time. Interestingly, TGF-β levels showed a rather large variability around steel on day 4 compared to Teflon. IL-10 gene expression remains high throughout wear time which can be explained by the fact that there is a constant release of reactive oxygen species and degradative enzymes in the cannula’s microenvironment, and therefore IL-10 cannot execute its full potential as an anti-inflammatory cytokine^39^.

The lack of insulin infusion presents the main limitation of this study, since there is an undoubted need for linking catheter longevity, immune response and glycemic control. The effect of insulin has previously only been studied over a couple of hours after catheter insertion^37,40^. Therefore, the question, to what extent insulin infusion influences the inflammatory response and catheter longevity, remains unanswered thus far. Since the methods used in this study require large amounts of tissue material, we studied the effects of catheter implantation in an animal model with similar skin and adipose tissue structure to humans^41^. Although the pig has proven to be an excellent model to study adipose tissue inflammation, the reaction may be somewhat different in humans^41,42^. Concepts of tissue explantation in humans have been proposed, but they raise ethical and methodological limitations. We experienced kinking of 2 Teflon and 2 steel cannulas during our study, all of which did not result in an occlusion alarm of the pump. Reasons for kinking of the steel cannula could be the considerable amount of force needed to insert the cannula through the medical adhesive and/or the rather rigid swine skin.

Insulin infusion sets are recommended to be changed every 2 to 3 days. In this study set-up, we chose 4 days of wear time to increase the likelihood of seeing a difference between the two cannula materials. We extended the wear time to 7 days, to systematically assess the time-dependent inflammatory response in an adequate animal model. This is of special of interest in the development of closed-loop artificial pancreas systems where continuous glucose monitoring devices have a longevity of 7 days^43^. In recent years, attempts have been made to combine both glucose sensor and insulin delivery in one device (“single-port”), reducing the burden of multiple insertions for a functioning AP system^44–48^. This is of special interest for children with small body surface areas for the insertion of CSII catheters and sensors. In order to combine CSII and continuous glucose monitoring in one device and to reduce the lifelong burden and the number of inserted catheters in a lifetime, the longevity of CSII catheters has to be increased substantially.

The quantitative as well as the qualitative histopathologic evaluation, gene expression analysis and the subjective observations during tissue excision did not suggest a better tolerability of flexible Teflon cannulas with a blunt end over rigid steel cannulas with a sharp tip in terms of tissue histology. However, an increased expression of pro-inflammatory cytokines after 7 days of wear-time was observed. The sharp tip and the stiffness of the steel cannula caused more bleeding in the first 4 days of wear-time.

Our study design was robust enough to show temporal changes in inflammation caused by a CSII catheter and thus presents a possibility for a standardised method assessing tissue-device interfaces systematically. The combination of different methods assessing the tissue reaction over time is a major strength of this study. The results of the quantitative and qualitative histopathological assessment and the gene expression analysis confirm each other and underline the robustness of the methods applied. Furthermore, each animal served as its own control, making the results more reliable despite the small sample size. Although some aspects cannot be directly translated from swine to humans, this study design serves as a strong and robust tool for further studies. Our study setup can be directly transferred and applied for any implantable device to generate more data on tissue response to materials considered biocompatible and safe by regulatory bodies.

Interestingly, cannula materials remain fairly unregulated for market entry, while insulin formulations are heavily regulated. In-depth studies of the inflammatory response to CSII catheters, not only assessing more time points but also including insulin infusion, should be considered to create a complete picture of the impact of cannula material and design on tolerability and longevity of CSII catheters. This information could be crucial for future recommendations for catheter use and catheter design, such as the development of anti-inflammatory coatings for cannulas, regulating cell and complement attachment.

## Conclusions

Both steel and Teflon are routinely used in clinical practice independent of their drawbacks which include bleeding for steel and kinking for Teflon^6,8^. The results of this study show that there was no superiority of one material over the other in terms of inflammation. However, a final statement can only be made when insulin infusion is added to the study setup. To our knowledge, this is the first systematic study to assess the difference in inflammatory response to commercially available steel and Teflon CSII catheters over wear-time.
