# Author Correction: Systematic *in vivo* evaluation of the time-dependent inflammatory response to steel and Teflon insulin infusion catheters

**DOI:** 10.1038/s41598-019-40316-z

**Published:** 2019-04-11

**Authors:** Jasmin R. Hauzenberger, Julia Münzker, Petra Kotzbeck, Martin Asslaber, Vladimir Bubalo, Jeffrey I. Joseph, Thomas R. Pieber

**Affiliations:** 1 Department of Internal Medicine, Division of Endocrinology and Diabetology, Medical University of Graz, Graz, Austria; 2 Institute of Pathology, Medical University of Graz, Graz, Austria; 3 Division of Biomedical Research, Medical University of Graz, Graz, Austria; 4 Department of Anesthesiology, Jefferson Artificial Pancreas Center, Thomas Jefferson University, Philadelphia, PA USA

Correction to: *Scientific Reports* 10.1038/s41598-017-18790-0, published online 18 January 2018

This Article contains typographical errors in the Acknowledgements section.

“Jasmin Hauzenberger was funded by the Medical University of Graz through the PhD Program Molecular Fundamentals of Inflammation (DK-MOLIN) as well as the BOSCH-Forschungsstiftung (Essen, Germany)”.

should read:

“Jasmin Hauzenberger was funded by the Austrian Science Fund (FWF) through the PhD program Molecular Fundamentals of Inflammation (DK-MOLIN, W1241) as well as the BOSCH-Forschungsstiftung (Essen, Germany)”.

